# Clinical efficacy of Chinese herbs for supplementing qi and activating blood circulation combined with N-acetylcysteine in the treatment of idiopathic pulmonary fibrosis: A systematic review and network meta-analysis

**DOI:** 10.1371/journal.pone.0265006

**Published:** 2022-03-04

**Authors:** Qinglu Pang, Guodong Li, Fang Cao, Haoge Liu, Wan Wei, Yang Jiao

**Affiliations:** 1 Department of Internal Medicine of Traditional Chinese Medicine, Beijing University of Chinese Medicine, Beijing, China; 2 Department of Respiratory, Beijing Changping Hospital of Integrated traditional Chinese and Western Medicine, Beijing, China; 3 Department of Geriatrics, Dongfang Hospital Affiliated to Beijing University of Chinese Medicine, Beijing, China; 4 Department of Respiratory, Dongfang Hospital Affiliated to Beijing University of Chinese Medicine, Beijing, China; The University of Mississippi Medical Center, UNITED STATES

## Abstract

**Background:**

Chinese herbs for supplementing qi and activating blood circulation (CH) combined with N-acetylcysteine (NAC) is widely used for idiopathic pulmonary fibrosis (IPF) in China, but there is a lack of literature to evaluate its efficacy and clinical value.

**Purpose:**

This study compared CH + NAC with other treatments by network meta-analysis to clarify its clinical value.

**Methods:**

Cochrane Library, PubMed, Embase, Web of Science, China National Knowledge Infrastructure, WanFang Data, VIP Database, and China Biology Medicine were searched. Outcomes included lung function (DLCO (%), VC (%), FVC (%), FVC (L)), 6-min walking distance (6MWD), score of St George’s respiratory questionnaire (SGRQ), blood gas analysis (PaO2, PaCO2). The data were analyzed by Review Manager 5.4, Stata 12.0 and ADDIS 1.16.5.

**Results:**

23 studies including 1390 patients (702 in intervention group and 688 in control group) were collected to compare 8 outcome indicators among different treatments involving CH, CH+NAC, CH+PFD, NAC, PFD and PFD+NAC on IPF. Network meta-analysis showed that CH was better than NAC in terms of DLCO (%) (MD = 5.14, 95%CI: 1.01 to 8.68) and 6MWD (MD = 49.17, 95%CI: 25.97 to 71.36) as well as PFD + NAC was better than NAC in terms of FVC (L) (MD = -0.56, 95%CI: -0.83 to -0.31). In rankings results, CH + NAC is the best in terms of FVC (%), SGRQ, PaO2 and PaCO2; CH is the best in terms of DLCO (%), VC (%) and 6MWD; CH + PFD is the best in terms of FVC (L).

**Conclusion:**

CH related treatments may have advantages in the treatment of IPF and CH + NAC may have clinical application value. However, limited by the quality and quantity of researches included, more rational and scientific randomized controlled trials containing large sample sizes need to be conducted to further verify our conclusions.

## Introduction

Idiopathic pulmonary fibrosis (IPF) is a chronic, progressive and fatal disease, which could lead to decreased lung compliance, impaired lung function, respiratory failure and death. The median survival time of the patients with IPF after diagnosis is 2–4 years [1]. What is worse, the pathogenesis of IPF is still unclear, and the specific therapeutic drug remains to be found.

Oxidative stress is widely considered as one of the most important mechanisms of IPF. Experimental reports in vivo and vitro have shown that N-acetylcysteine (NAC), an antioxidant drug, could improve pulmonary fibrosis [2, 3]. However, clinical studies indicated that the efficacy of NAC for IPF is controversial. Maurits et al. demonstrated that NAC could have some beneficial effects on percentage of predicted carbon monoxide diffusing capacity (DLCO (%)) in the patients with IPF [4]. The results of study carried out by Homma et al. showed that percentage of predicted vital capacity (VC (%)) in the patients with IPF could also be ameliorated by NAC [5]. Nevertheless, Tomioka et al. indicated that there were no significant differences observed in pulmonary function, 6-min walking distance (6MWD) or quality of life between patients treated with NAC or placebo [6]. Nintedanib and pirfenidone (PFD) are the only two known drugs which are conditionally recommended for the treatment of IPF by the Food and Drug Administration (FDA) [7]. Clinical data, from several randomized, placebo-controlled, phase III trials, have shown that PFD, as an oral pyridine with antifibrotic, anti-inflammatory and antioxidant functions, could reduce decline in vital capacity (VC)/forced vital capacity (FVC), IPF-related and all-cause mortality of patients with IPF [8–11]. Nintedanib, a tyrosine kinase inhibitor with antifibrotic properties, has also been indicated to improve FVC in patients with IPF [12]. A meta-analysis showed that PFD and Nintedanib were better than NAC [13]. Despite all this, they are still limited in the treatment of IPF due to the high price and side effects [14, 15]. Therefore, seeking for a better treatment is an important issue to be solved urgently.

As a unique medical treatment, Chinese herbs which have been used to treat disease in China under the guidance of traditional Chinese medicine theory for thousands of years, have displayed its advantages in the treatment of IPF through a multi-level and multi-target approach [16]. According to the theory of traditional Chinese medicine, qi deficiency and blood stasis are the main pathogenesis of IPF. Therefore, supplementing qi and activating blood circulation should be the main treatment principle for IPF. Clinical researches demonstrated that the efficacy of Chinese herbs for supplementing qi and activating blood circulation (CH), or CH combined with NAC (CH+NAC) is better than NAC alone in improving outcomes including DLCO (%), VC (%), percentage of predicted forced vital capacity (FVC (%)), FVC (L), 6MWD, score of St George’s respiratory questionnaire (SGRQ) of IPF [17–23]. However, due to the lack of comparative research data between CH, CH + NAC, PFD or nintedanib in the treatment for IPF, we do not know whether it is necessary to combine CH with NAC.

Network meta-analysis (NMA), a development of traditional meta-analysis, could simultaneously compare multiple different interventions with each other and analyze the results of direct and indirect comparisons [24, 25]. In this study, we searched the electronic database, established a Bayesian network meta-analysis model, and tried to compare the efficacy of CH, CH + NAC, NAC, PFD, nintedanib, or other related treatments for IPF to clarify the clinical application value of CH + NAC in the treatment for IPF.

## Materials and methods

### Inclusion criteria

All studies were in English or Chinese. The experimental study should meet the following three conditions:

1. All patients were diagnosed with IPF. The diagnostic criteria were in accordance with guidelines developed by Chinese Medical Association, American Thoracic Society/European Respiratory Society (ATS/ERS) or American Thoracic Society/European Respiratory Society/Japanese Respiratory Society/Latin American Thoracic Association (ATS/ERS/JRS/ALAT) [26–31];
2. All studies included were randomized controlled trials (RCTs). All patients received necessary treatment, including oxygen inhalation, anti-infection and so on. Interventions included CH, CH+NAC, PFD, nintedanib and other related treatments. The control group included NAC, PFD or nintedanib. The duration of trials should be limited to 3–6 months (or 12–24 weeks).
3. Outcome indicators included lung function (including DLCO (%), VC (%), FVC (%), FVC (L)), 6MWD, SGRQ, blood gas analysis (including partial pressure of oxygen in arterial blood (PaO2), partial pressure of carbon dioxide in arterial blood (PaCO2)).

### Exclusion criteria

Studies are excluded if they meet one of the following conditions:

1. The patients in study were not diagnosed with IPF;
2. experimentation on animals or in vitro;
3. incomplete or incorrect data;
4. the full text of the study was not available;
5. Chinese herbs in the study could not supplement qi or activate blood circulation;
6. interventions involving non-oral drugs or acupuncture.

### Search strategy

Eight electronic databases including the Cochrane Library, PubMed, Embase, Web of Science, China National Knowledge Infrastructure (CNKI), WanFang Data, VIP Database, and China Biology Medicine (CBM) were searched. Studies we searched in each database were published from the inception of each database to January 29th, 2021. The search was conducted by the combination of subject words and free words. Search terms included "Idiopathic Pulmonary Fibrosis", "IPF", "Pulmonary Fibrosis", "Lung Fibrosis", "Traditional Chinese Medicine", "Drugs Chinese Herbal", "TCM", "Chinese Medicine", "Herbal Medicine", "Herbal Drug", "Decoction", "Herb", "Pirfenidone", "PFD", "Nintedanib".

### Data extraction

All included studies were imported into EndNote X9 (developed by Clarivate Analytics (US) LLC) software (version 9.3.3) and screened by two independent researchers (CF and LHG). If there was a disagreement, a third researcher would resolve it (JY). The information and data of the included studies were collected as follows: (1) the name of the first author and the year of publication, (2) the number of patients and their gender and age, (3) the intervention and duration of it, (4) outcome indicators including DLCO (%), VC (%), FVC (%), FVC (L), 6MWD, SGRQ, PaO2, PaCO2.

### Risk of bias within studies

The Cochrane risk of bias (ROB) assessment tool was used to evaluate the quality of the included studies [32]. This assessment includes seven parts: (1) random sequence generation, (2) concealment of the distribution plan, (3) blinding of subjects and researchers, (4) blinding of the evaluators to the outcome, (5) the integrity of the final data, (6) selective reporting of research results, (7) other sources of bias. Three evaluation levels including low risk, unclear, and high risk were applied to assess the seven parts. Two independent investigators (CF and LHG) assessed the quality of the included RCTs; if there was a disagreement, a third researcher (JY) would resolve it.

### Statistical analysis

In terms of statistical analysis, we partly referred to Lu’s method [33]. Review Manager 5.4 (developed by Cochrane Collaboration), STATA 12.0 (developed by StataCorp LLC) and Aggregate Data Drug Information System 1.16.5 (ADDIS, developed by Innovative Medicines Initiative and UMCG) were used for statistical analysis. In this study, odds ratio (OR) with 95% confidence interval (CI) were used as the effect for dichotomous data while mean difference (MD) for continuous variable. Review Manager 5.4 was used for literature quality evaluation. Stata 12.0 was used to draw the network plot, evaluate the heterogeneity, and comparison-adjusted funnel plot of each outcome. The heterogeneity was assessed by the value of I^2^ according to the method of Higgins et al. [34]. When I^2^>50%, the heterogeneity of the data is considered to be significant, otherwise, the heterogeneity is considered to be moderate (25%<I^2^<50%) or small (I^2^<25%). In the network graph, the size of treatment nodes reflected the number of patients randomly allocated to each treatment, and the thickness of edges represented the number of studies in each comparison.

ADDIS 1.16.5, based on the Bayesian framework using the Markov chain Monte Carlo (MCMC) method for prior evaluation and implementation, was used for the network meta-analysis. Four Markov chains were used to set the initial value. The variance scaling factor of the model was 2.5, the thinning interval was 10, the tuning iterations were 20000, and the simulation iterations were 50000. When the potential scale reduced factor (PSRF) tends to 1, the convergence degree was satisfied. Finally, the results of network meta-analysis were presented in tabular form, and the probability of each intervention becoming the best one was offered by ranking probability.

## Results

### Literature search results

**Fig 1:**
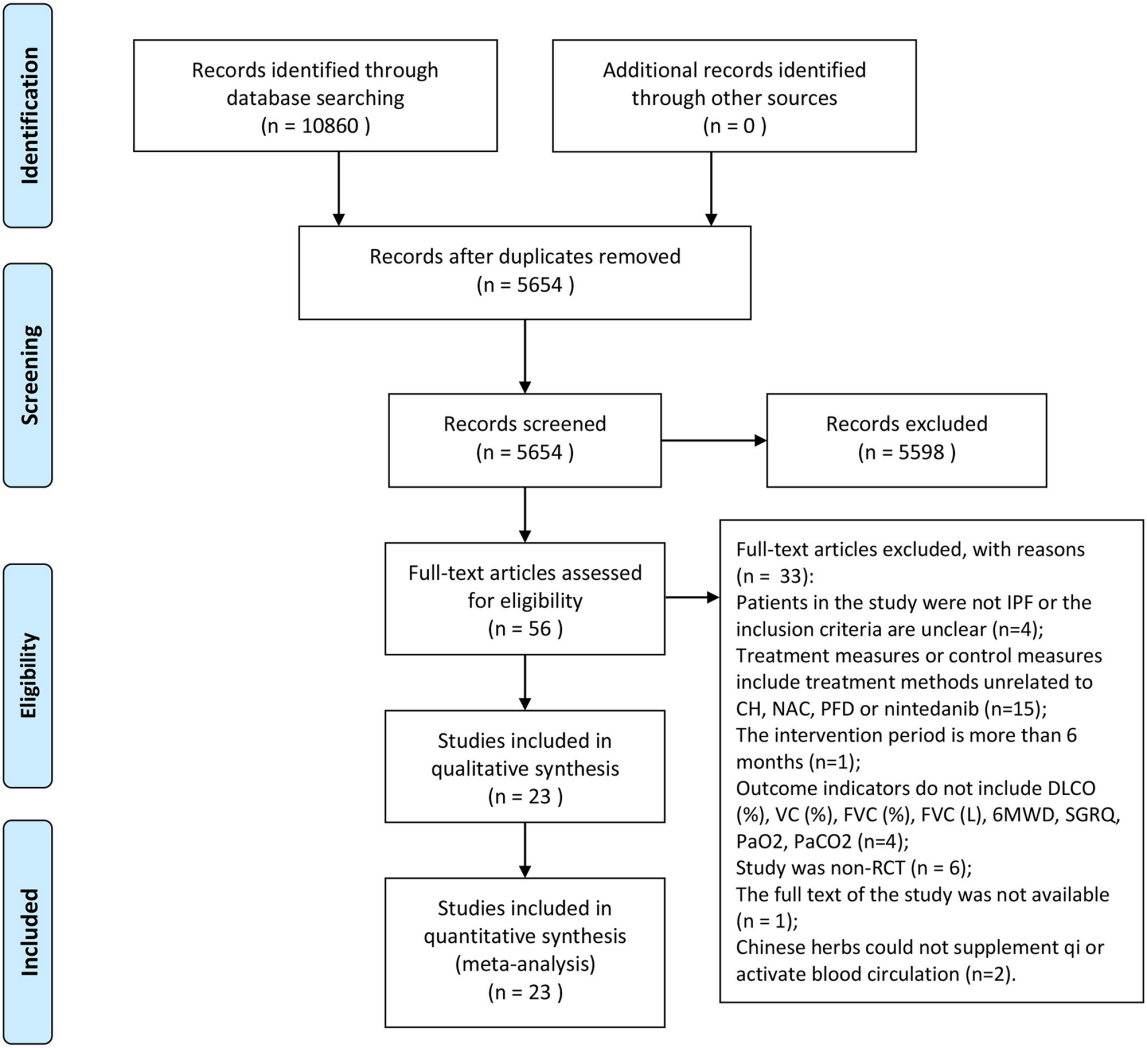
Flow chart of study selection.

This study obtained 10860 articles totally in the initial examination according to the search strategy. Then we deleted 5206 duplicate records and 5654 studies were left for further screen. After reading the title and abstract, 5598 studies were excluded. By examining 56 full-text articles, we excluded 33 ones. The reasons that they were excluded were as follows: Patients in the study were not IPF or the inclusion criteria was unclear (n = 4); Treatment measures or control measures included methods unrelated to CH, NAC, PFD or nintedanib (n = 15); The intervention period was more than 6 months (n = 1); Outcome indicators did not include DLCO (%), VC (%), FVC (%), FVC (L), 6MWD, SGRQ, PaO2, PaCO2 (n = 4); Study was non-RCT (n = 6); The full text of the study was not available (n = 1); Chinese herbs could not supplement qi or activate blood circulation (n = 2). Finally, 23 articles [17–23, 35–50] were included for the network meta-analysis. The process of literature selection and exclusion was shown in Fig 1.

### Study characteristics

**Fig 2:**
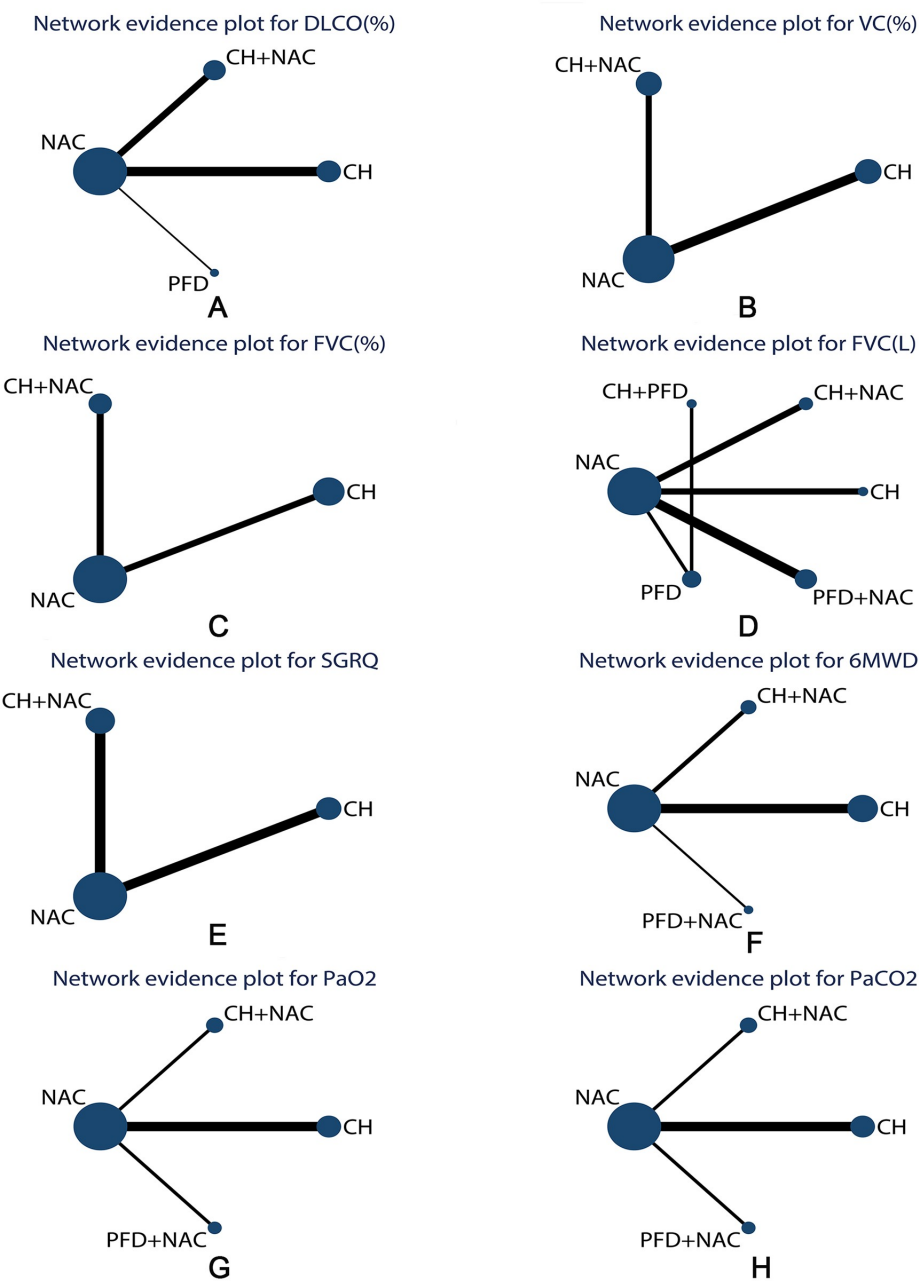
Network plots for the 8 outcomes. A: DLCO (%). B: VC (%). C: FVC (%). D: FVC (L). E: 6MWD. F: SGRQ. G: PaO2. H: PaCO2. CH, Chinese herbs for supplementing qi and activating blood circulation; CH + NAC, CH combined with N-acetylcysteine; CH + PFD, CH combined with pirfenidone; NAC, N-acetylcysteine; PFD, pirfenidone; PFD + NAC, pirfenidone combined with N-acetylcysteine.

23 studies were published in 2012–2020. Among them, 3 RCTs were published in 2012–2015 and 20 in 2016–2020. Of the 23 articles, 1411 patients were involved, including 720 in the intervention group and 691 in the control group. There were 857 men, accounting for 61% of the total number, and 554 women, accounting for 39% of the total number. The mean age of the patients included in this study ranged from 46.03 to 68.5 years. About the course of treatment, 7 RCTs were 6 months, 2 were 4 months and 14 were 3 months (or 12 weeks). All the subjects were from China and all baseline data were comparable. There were 6 treatments involved, including CH, CH + NAC, CH + PFD, NAC, PFD, PFD + NAC. There were 10, 7, 5, 9, 9, 14, 4 and 4 studies reported the DLCO (%), VC (%), FVC (%), FVC (L), SGRQ, 6MWD, PaO2, PaCO2, respectively. The network plots of 8 outcomes was presented in Fig 2. Network plots which were less than three studies were not presented in the study. Details of the characteristics of the studies were shown in Table 1.

**Table 1:** Details of the characteristics of the studies.

ID	Gender (M/F)	Age (E/C)	Sample size (E/C)	Treatment (E/C)	Comparability of baseline data	Course	Outcome
Fan M, 2012	26/17	60.98±9.22/65.12±8.61	43 (22/21)	CH/NAC (0.2g tid)	YES	3 months	① ② ⑥
Li Z, 2015	32/32	58.23±8.35/59.98±7.43	64 (34/30)	CH/NAC (0.6g qd)	YES	3 months	① ② ⑥
Chen H, 2016	17/9	60.4±5.9/59.7±6.3	26(13/13)	CH/NAC (0.6g qd)	YES	3 months	① ② ⑥
Li Y, 2016	26/22	61.88±6.12/62.63±5.69	48(24/24)	PFD (0.6g tid) + NAC (0.6g tid) /NAC (0.6g tid)	YES	6 months	④ ⑥ ⑦ ⑧
Wang Q, 2016	42/38	60.51±6.82/59.23±6.91	80(43/37)	CH+NAC (0.6g tid) /NAC (0.6g tid)	YES	3 months	① ②
Xin D, 2016	29/20	65.68±7.5/65.88±7.51	49 (25/24)	CH/NAC (0.2g tid)	YES	3 months	⑤ ⑥
Miao Q, 2017	35/20	62.36±10.4/65.68±10.05	55 (28/27)	CH+NAC (0.2g tid) /NAC (0.2g tid)	YES	3 months	① ② ⑤ ⑥
Feng J, 2018	35/27	61.0±10.1/62.3±8.5	62 (31/31)	CH/NAC (0.6g tid)	YES	6 months	④ ⑥
Gu C, 2018	34/26	62.3±7.6/63.8±8.5	60 (30/30)	CH+PFD (0.6g tid) /PFD (0.6g tid)	YES	6 months	③ ⑤
Li N, 2018	28/22	63.27±8.34/66.23±5.48	50 (25/25)	CH+NAC (0.2g tid) /NAC (0.2g tid)	YES	12 weeks	① ③ ⑥
Liang C, 2018	53/32	46.03±5.17/47.06±6.05	85 (42/43)	CH/NAC (0.6g tid)	YES	6 months	③
Mao Z, 2018	43/24	58.62±13.99	67 (32/35)	PFD (0.6g tid) + NAC (0.2g tid) /NAC (0.2g tid)	YES	4 months	④
Xi N, 2018	38/22	65.11±7.46/64.28±7.15	60 (30/30)	CH+NAC (0.6g tid) /NAC (0.6g tid)	YES	3 months	① ② ⑤ ⑥ ⑦ ⑧
Zhang H, 2018	34/14	52.7±7.2/51.7±6.9/53.9±6.8	48 (18/15/15)	CH/CH/NAC (0.2g tid)	YES	3 months	① ② ⑥ ⑦ ⑧
Zhao G, 2018	34/26	58/59	60 (30/30)	CH/NAC (0.6g tid)	YES	3 months	① ③ ⑤ ⑥
Bai W, 2019	51/33	56.29±6.53/56.48±6.71	84 (42/42)	CH+PFD (0.2g tid) /PFD (0.2g tid)	YES	3 months	④
Guo H, 2019	49/33	59.57±11.33/59.44±12.45	82 (41/41)	PFD (0.6g tid) +NAC (0.2g tid) /NAC (0.2g tid)	YES	4 months	④
Wen J, 2019	47/39	56.24±10.24/55.63±10.54	86 (43/43)	PFD (0.4g tid) /NAC (0.6g tid)	YES	6 months	① ④
Yang S, 2019	39/33	64.53±5.4/65.37±5.7	72 (36/36)	CH/NAC (0.6g tid)	YES	3 months	⑤ ⑥
Jiang H, 2020	19/11	50.1±6.2/48.5±5.8	30 (15/15)	CH/NAC (0.3g tid)	YES	3 months	④ ⑤ ⑦ ⑧
Meng Y, 2020	29/31	63.1±8.2/62.8±5.5	60 (30/30)	CH+NAC (0.2g tid) /NAC (0.2g tid)	YES	6 months	③ ④ ⑤
Xue H, 2020	46/18	68.5±7.5/66.8±8.6	64 (33/31)	CH+NAC (0.6g tid) /NAC (0.6g tid)	YES	3 months	④ ⑤ ⑥
Huang H, 2015	71/5	59.03±5.94/61.61±6.39	76 (38/38)	PFD (0.6g tid) +NAC (0.6g tid) /NAC (0.6g tid)	YES	6 months	⑥

^①^: DLCO (%);

^②^: VC (%);

^③^: FVC (%);

^④^: FVC (L);

^⑤^: 6MWD;

^⑥^: SGRQ;

^⑦^: PaO2;

^⑧^: PaCO2.

### Risk of bias within studies

**Fig 3:**
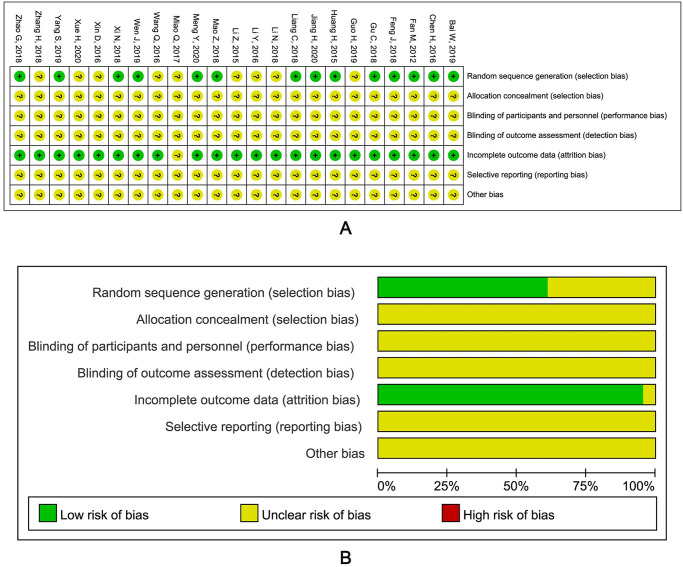
Assessment of risk of bias.

Among the 23 RCTs, 14 [17–21, 35–37, 39, 42, 45–46, 49, 50] of them which mentioned the specific random allocation method were considered as “low-risk”, and the other 9 [22, 23, 38, 40, 41, 43, 44, 47, 48] were considered as “unclear”. None of 23 RCTs mentioned allocation concealment, blinding of subjects and researchers or blinding of the evaluators to the outcome, so they were all evaluated as "unclear" in the three parts. Most of the RCTs reported complete outcome data and were regarded as “low risk of bias” while the other one [43] was “unclear” in terms of the incomplete outcome data. About the rest two parts, selective reporting results and other bias, detailed information cannot be obtained, thus we evaluated all of them as "unclear". The specific results of bias risk assessment for all RCTs were shown in Fig 3.

### Network meta-analysis and rank probability

#### Lung function

**Fig 4:**
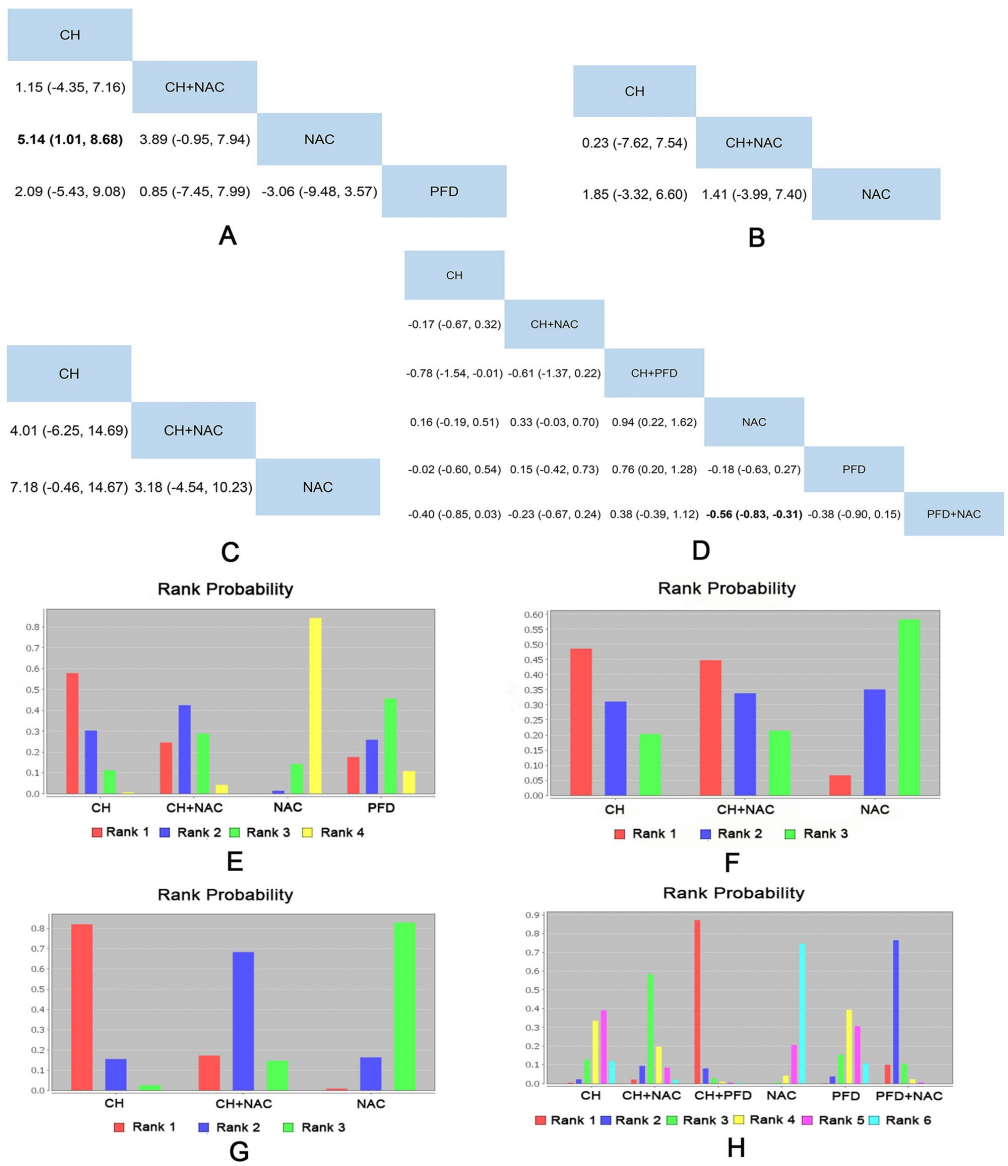
Network meta-analysis and rank probability of lung function. A: Network Meta-analysis of DLCO (%). B: Network Meta-analysis of VC (%). C: Network Meta-analysis of FVC (%). D: Network Meta-analysis of FVC (L). E: Rank Probability of DLCO (%). F: Rank Probability of VC (%). G: Rank Probability of FVC (%). H: Rank Probability of FVC (L).

10 RCTs including 4 interventions (CH, CH + NAC, NAC and PFD), 7 RCTs including 3 interventions (CH, CH + NAC and NAC), 4 RCTs (the other 1 was not involved in this network) including 3 interventions (CH, CH + NAC and NAC) and 9 RCTs including 6 interventions (CH, CH + NAC, CH + PFD, NAC, PFD and PFD + NAC) reported the DLCO (%), VC (%), FVC (%) and FVC (L) as outcome indicator respectively. The results only showed that CH was better than NAC (MD = 5.14, 95%CI: 1.01 to 8.68) in terms of DLCO (%) as well as PFD + NAC was better than NAC (MD = -0.56, 95%CI: -0.83 to -0.31) in terms of FVC (L). Detailed results were shown in Fig 4A–4D.

The rank probability of each intervention was represented in Fig 4E–4H. In terms of these four outcomes, rank 1 was the best intervention, and rank N was worst. About DLCO (%), the rank of interventions was as follows: CH (5 RCTs) > CH + NAC (4 RCTs) > PFD (1 RCTs) > NAC (10 RCTs). About VC (%), the rank of interventions was as follows: CH (4 RCTs) > CH + NAC (3 RCTs) > NAC (7 RCTs). About FVC (%), the rank of interventions was as follows: CH + NAC (2 RCTs) > CH (2 RCTs) > NAC (4 RCTs). About FVC(L), the rank of interventions was as follows: CH + PFD (1 RCTs) > PFD + NAC (3 RCTs) > CH + NAC (2 RCTs) > PFD (2 RCTs) > CH (2 RCTs)>NAC (8 RCTs). Specific data were shown in S1 Table.

In terms of DLCO (%), the data of comparison between CH and NAC showed moderate heterogeneity (I^2^ = 36.4%), while the data of comparison between CH + NAC and NAC showed significant heterogeneity (I^2^ = 52.9%) (seen in S1 Fig). In terms of VC (%) and FVC (%), the data showed small heterogeneity (seen in S2 and S3 Figs respectively). In terms of FVC (L), the data of comparison between NAC and NAC + PFD showed significant heterogeneity (I^2^ = 52.5%), while the others showed small heterogeneity (seen in S4 Fig).

#### SGRQ

**Fig 5:**
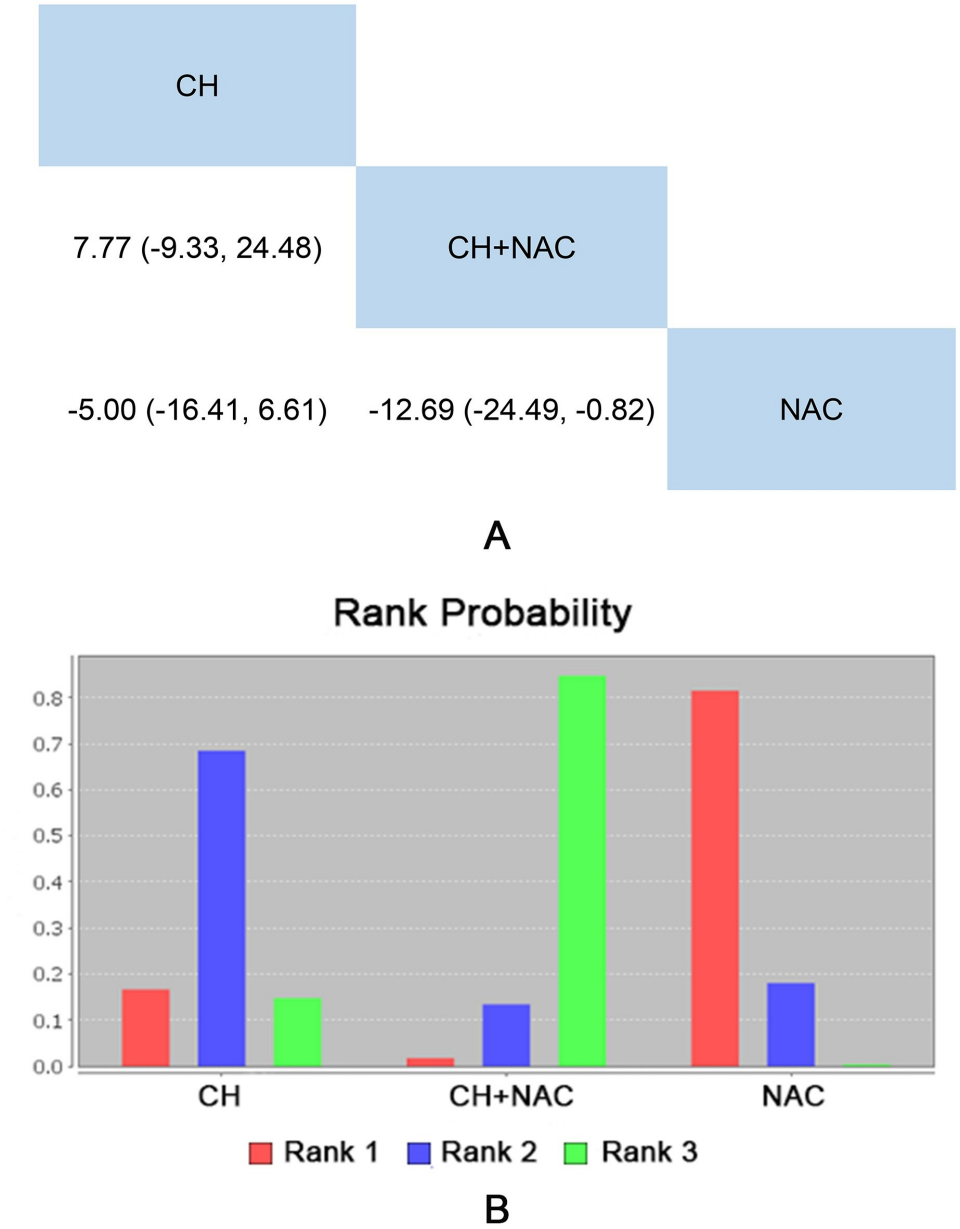
Network meta-analysis and rank probability of SGRQ. A: Network Meta-analysis of SGRQ. B: Rank Probability of SGRQ.

8 RCTs (the other 1 was not involved in this network) including 3 interventions (CH, CH + NAC and NAC) reported the SGRQ as outcome indicator. The results showed that there was no significant difference among all treatments. Detailed results were shown in Fig 5A.

The rank probability of each intervention was represented in Fig 5B. In terms of SGRQ, rank 1 was the worst intervention, and rank N was best. The rank of interventions was as follows: NAC (8 RCTs) > CH (4 RCTs) > CH + NAC (4 RCTs). Specific data were shown in S1 Table.

Significant heterogeneity (I^2^_CH vs NAC_ = 81.1%, I^2^_CH + NAC vs NAC_ = 86.1%) existed in the data of comparison between CH and NAC as well as CH + NAC and NAC (seen in S5 Fig).

#### 6MWD

**Fig 6:**
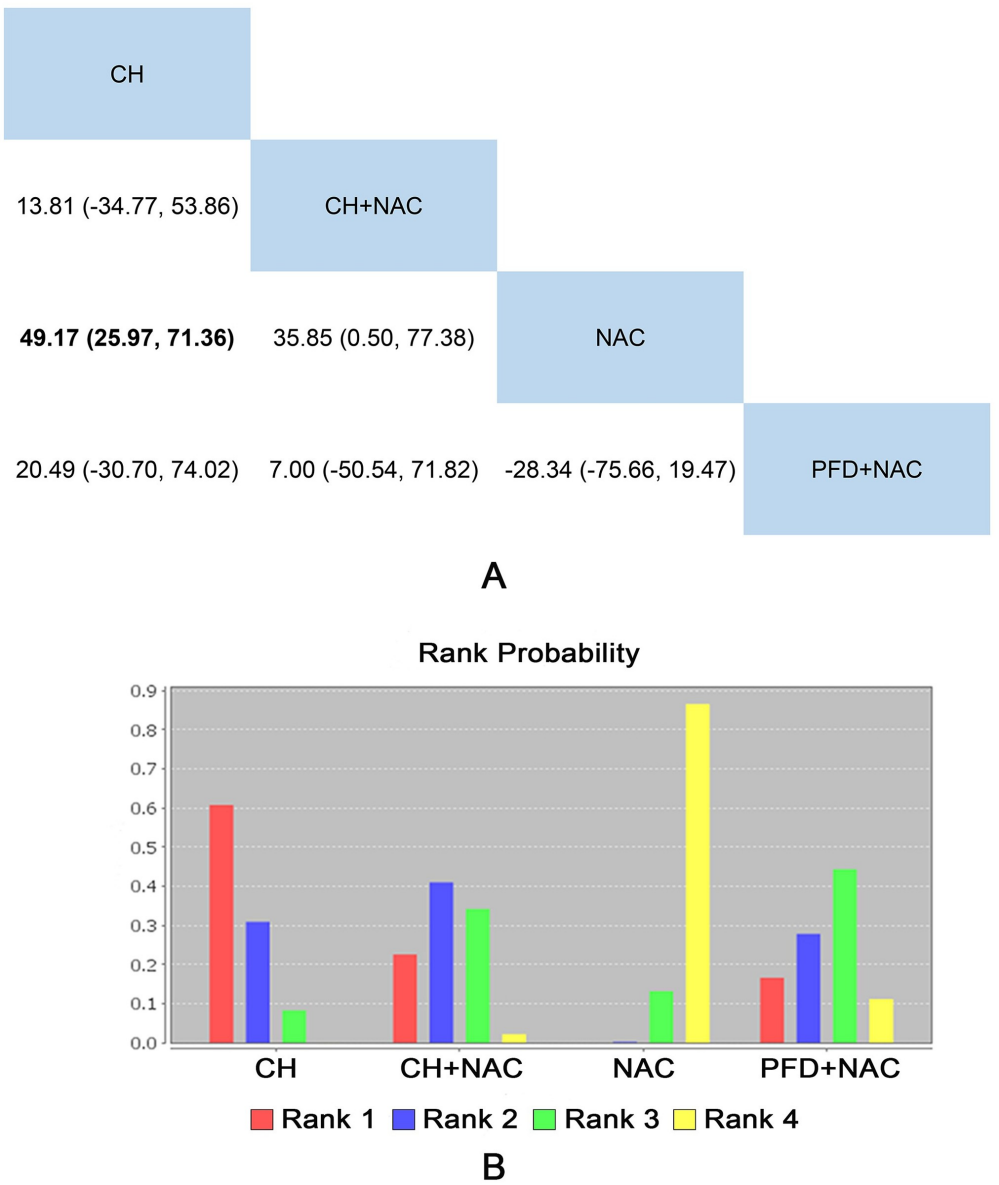
Network meta-analysis and rank probability of 6MWD. A: Network Meta-analysis of 6MWD. B: Rank Probability of 6MWD.

14 RCTs including 4 interventions (CH, CH + NAC, NAC and PFD + NAC) reported the 6MWD as outcome indicator. The results only showed that CH was better than NAC (MD = 49.17, 95%CI: 25.97 to 71.36). Detailed results were shown in Fig 6A.

The rank probability of each intervention was represented in Fig 6B. The rank 1 was the best intervention, and rank N was worst. The rank of interventions was as follows: CH (8 RCTs) > CH + NAC (4 RCTs) > CH + PFD (2 RCTs) > NAC (14 RCTs). Specific data were shown in S1 Table.

Significant heterogeneity (I^2^_CH vs NAC_ = 96.3%, I^2^_NAC vs NAC+PFD_ = 93.9%) existed in the data of comparison between CH and NAC as well as NAC and NAC+PFD, while the data of comparison between CH + NAC and NAC showed small heterogeneity (seen in S6 Fig).

#### Blood gas analysis

**Fig 7:**
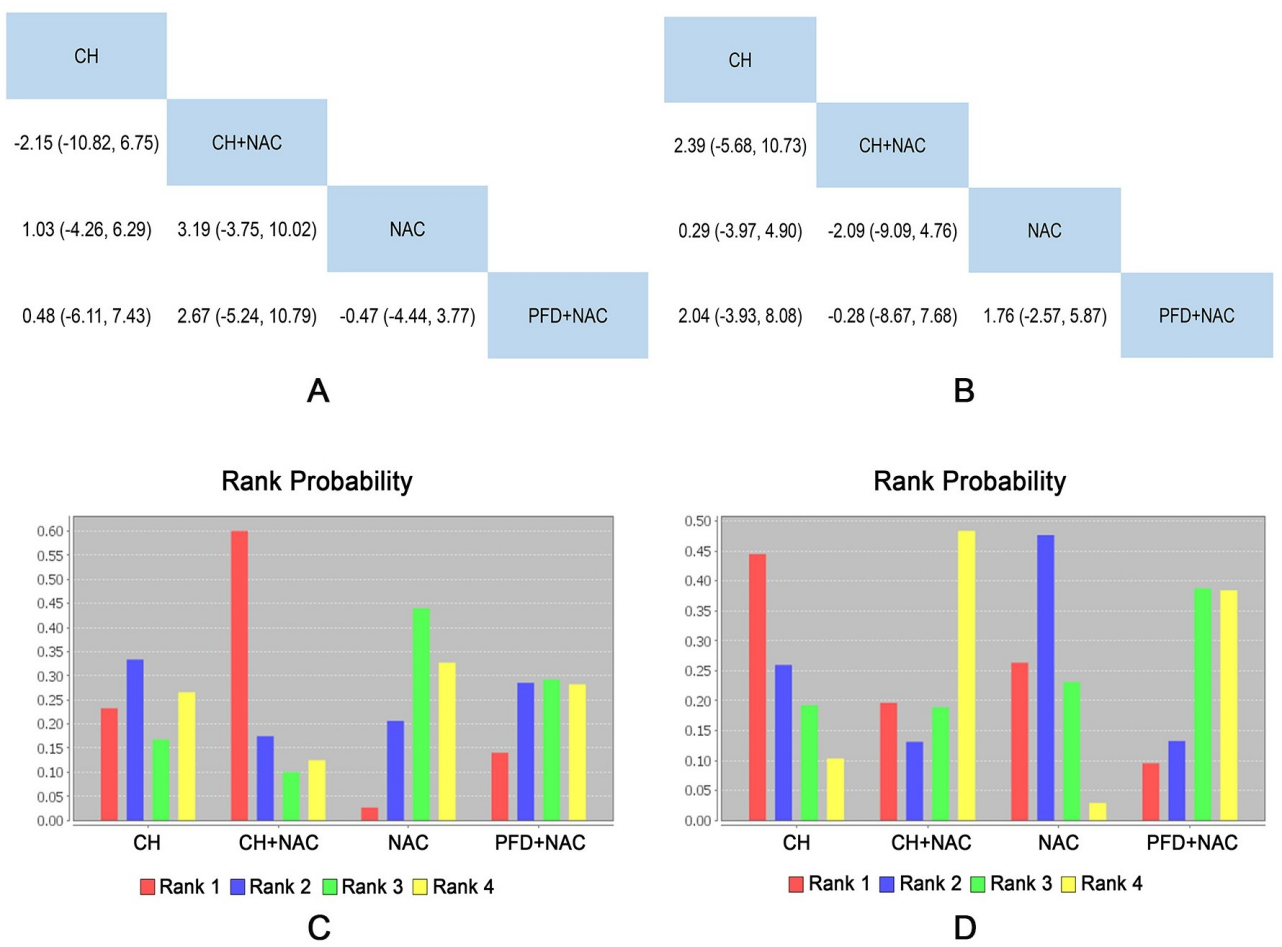
Network meta-analysis and rank probability of blood gas analysis. A: Network Meta-analysis of PaO2. B: Network Meta-analysis of PaCO2. C: Rank Probability of PaO2. D: Rank Probability of PaCO2.

4 RCTs including 4 interventions (CH, CH + NAC, NAC and PFD + NAC), reported the blood gas analysis (including PaO2, PaCO2) as outcome indicator. The results showed that there was no significant difference among all treatments. Detailed results were shown in Fig 7A and 7B.

The rank probability of each intervention was represented in Fig 7C and 7D. In terms of PaO2, rank 1 was the best intervention, and rank N was worst. The rank of interventions was as follows: CH + NAC (1 RCT) > CH (2 RCT) > NAC (4 RCTs) > PFD + NAC (1 RCT). In terms of PaCO2, rank 1 was the best intervention, and rank N was worst. The rank of interventions was as follows: CH (2 RCTs) > NAC (4 RCTs) > PFD + NAC (1 RCTs) > CH + NAC (1 RCTs). Specific data were shown in S1 Table.

In terms of PaO2 and PaCO2, the data showed small heterogeneity (seen in S7 and S8 Figs respectively).

### Publication bias

**Fig 8:**
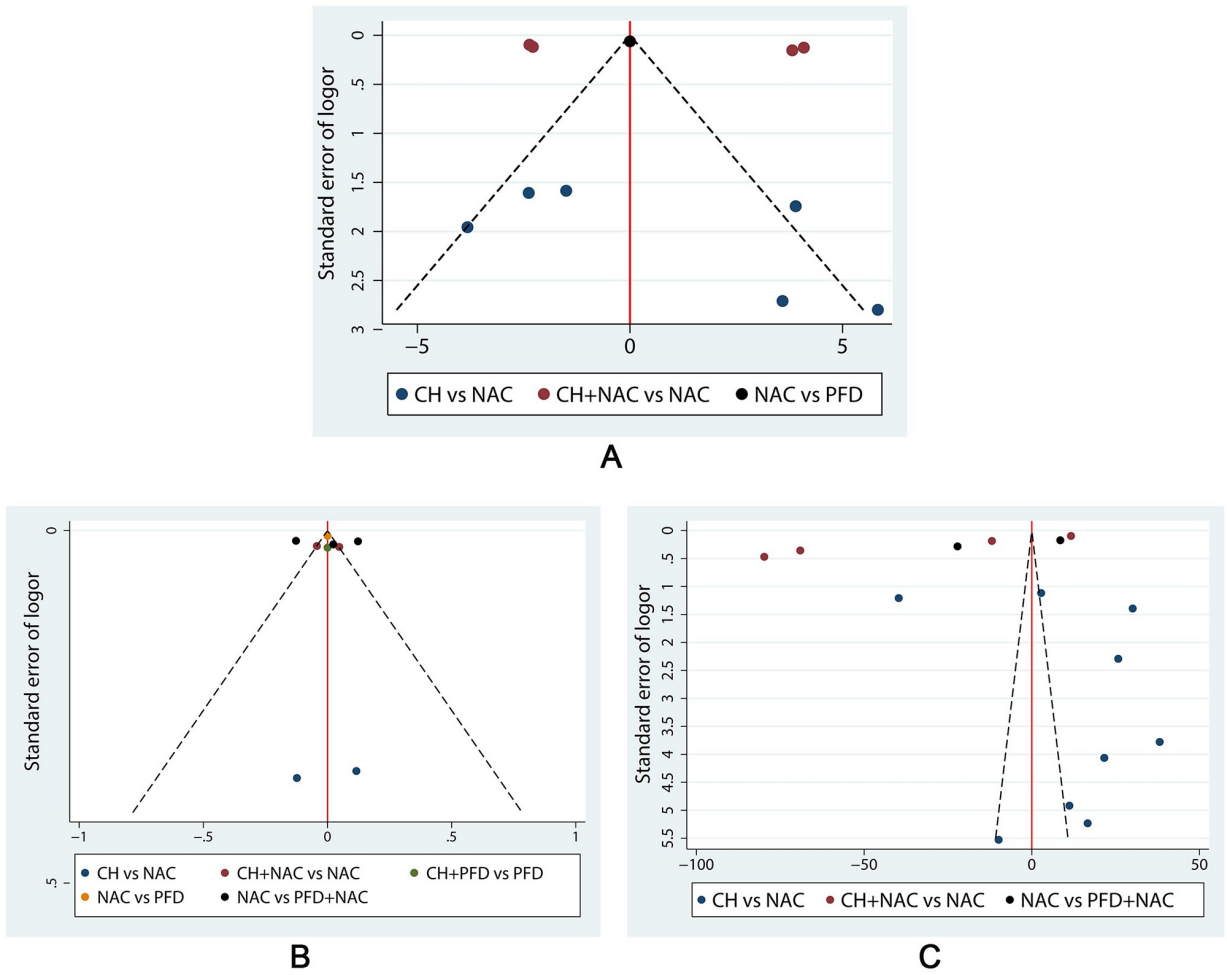
Publication bias. A: Comparison-adjusted funnel plot of DLCO (%). B: Comparison-adjusted funnel plot of FVC (L). C: Comparison-adjusted funnel plot of 6MWD.

Outcome indicators including at least 9 RCTs were analyzed for publication bias. Comparison-adjusted funnel plots of DLCO (%), FVC (L) and 6MWD were displayed in Fig 8. Differently colored points represented comparisons among the different interventions. Funnel plots of DLCO (%) and 6MWD were not quite symmetric, indicating potential publication bias in the network.

## Discussion

At present, lung transplantation, as the only radical treatment of IPF, could not be widely used in clinical treatment due to the lack of lung tissue source and postoperative immune rejection. Medical drugs are still not curative for IPF. Delaying lung function deterioration, improving quality of life and delaying disease progression are the goals of current pharmacotherapy. Nowadays, it is internationally recognized that PFD and nintedanib are the only two drugs which have certain efficacy on IPF although they are expensive and have certain side effects. In recent years, there have been more and more relevant studies on TCM treatment for IPF, and clinical studies and meta-analysis have shown that CH can improve clinical symptoms, delay the deterioration of lung function and improve the quality of life of patients with IPF [51, 52]. Experimental studies also showed that CH could significantly improve lung function, pathological products, as well as survival in animal models with pulmonary fibrosis [53]. The effect of NAC in the treatment of IPF is controversial, while CH + NAC is widely used for the treatment of IPF in China and the efficacy of it has been reported to be better than that of NAC alone. However, there is a lack of clinical studies that compared efficacy of CH with that of CH + NAC, as well as related meta-analysis. Therefore, we cannot know whether CH + NAC for treatment of IPF has practical significance for clinical application. In this study, we attempted to solve this problem by NMA and evaluate the efficacy of CH + NAC for IPF by contrast with PFD, nintedanib, or other related treatments. The main clinical manifestation of patients with IPF is progressive aggravation of dyspnea, restrictive ventilation dysfunction and gas-exchange disorder, hypoxemia and even respiratory failure [54]. And thus quality of life is also affected seriously. Lung function, PaO2, score of SGRQ and 6MWD are highly correlated with prognosis which are independent risk factors for death of patients with IPF [55–57]. Therefore, our study used lung function, SGRQ, 6MWD and blood gas analysis as outcome indicators.

In this study, we pooled data of 23 RCTs including 1390 patients to compare the effects among different treatments involving CH, CH+NAC, CH+PFD, NAC, PFD and PFD+NAC on patients with IPF. Unfortunately, according to inclusion and exclusion criteria, treatments related to nintedanib were not obtained. NMA showed that there were significant differences in terms of DLCO (%) and SGRQ between CH and NAC, while the others were not. Based on the ranking results, we found that CH + NAC was the best in terms of FVC (%), SGRQ, PaO2 and PaCO2; CH was the best in terms of DLCO (%), VC (%) and 6MWD; CH + PFD was the best in terms of FVC (L). That means treatments related to CH might have its advantages in the treatment of IPF. CH and CH + NAC ranked the top two in terms of DLCO (%), VC (%), FVC (%), SGRQ, 6WMD and PaO2. In addition, CH was better than CH + NAC in terms of DLCO (%), VC (%) and 6WMD, yet CH + NAC was better than CH in terms of FVC (%) and FVC (L), SGRQ, PaO2 and PaCO2. These results not only confirmed the advantages of these two treatments for IPF, but also provided some evidence for the rationality and clinical value of CH + NAC. In terms of FVC (L), CH + PFD was the best treatment followed by PFD + NAC, suggesting that CH, as an adjuvant treatment, had potential value as well as CH + PFD might be a better treatment. However, there was only 1 study involving CH + PFD, so that we could not confirm this result. In the rankings including PFD (DLCO (%), FVC (L)), the results showed that CH + NAC was better than PFD alone, which confirmed the clinical value of CH + NAC again. NAC ranked last two in all rankings, suggesting that NAC alone was indeed less effective in the treatment for IPF. Indicators related to PFD + NAC included FVC (L), PaO2 and PaCO2. It ranked second in FVC (L), but ranked relatively lower in other outcome indicators. Literature report showed that PFD + NAC could improve the decline of FVC (L) and 6WMD in patients with IPF, but it also suggested that the treatment scheme had side effects [52]. Therefore, based on the analysis of the above results, we believe that CH + NAC might be a promising treatment.

Significant heterogeneity exists in the results of DLCO (%), FVC (L), SGRQ, and 6MWD. This may affect the accuracy of the results. Due to the small number of samples, the condition of patients may be different between studies. That may cause heterogeneity. In addition, the heterogeneity of SGRQ and 6MWD may also come from the subjectivity of the measurers and the subjects. Even if these controversial results are removed, the main conclusion of this study can still be drawn.

At present, the pathogenesis of IPF is not clear. Oxidative stress, inflammation and epithelial mesenchymal transformation are considered to be the main pathogenesis of the disease. In recent years, there have been more and more studies on the mechanism of anti-fibrosis related to CH, which has attracted more and more attention in the field of treating pulmonary fibrosis. Yu et al. found that CH could improve conditions in rats with pulmonary fibrosis by relieving the histopathologic changes, improving ventilation function, compliance and work of breathing, meliorating the capacity and elasticity of the lungs, as well as stabilizing the alveolar surface tension [58]. An experimental study of Wang et al. suggested that CH could ameliorate the pulmonary fibrosis, the possible mechanism might involve inhibition of pulmonary inflammation and collagen deposition, possibly via suppressing TGF-β1/Smad3/PAI-1 signaling pathway [59]. It was also found that CH could ameliorate pulmonary fibrosis by inhibiting JAK-STAT signaling pathway [60]. Our results also suggested that CH related treatments might have advantages in the treatment of IPF, yet the potential molecular mechanism remains unclear. Therefore, further molecular and clinical researches are significant and necessary.

There have been studies which conducted meta-analysis to evaluate the treatment of IPF with traditional Chinese medicine related therapies. The research of Wu Q et al. [61] showed that traditional Chinese medicine related treatment had advantages over simple western medicine in the treatment of IPF. Studies of Zhou M et al. [52] and Zhang Y et al. [62] further demonstrated that the traditional Chinese medicine of Nourishing Yin, supplementing qi and activating blood circulation was better than simple western medicine for IPF. However, the control treatment scheme and traditional Chinese medicine treatment scheme of these studies were not clear, and relevant subgroup analysis was not conducted. That means these studies could only conclude that traditional Chinese medicine related treatment methods have certain advantages for IPF, but it is still not clear which specific clinical treatment scheme has more advantages. Meanwhile, these studies did not limit the intervention time included in the study. If the intervention cycle is quite different, it may cause methodological heterogeneity. IPF is a progressive disease, and thus the outcome indicators of short-term clinical research and long-term clinical research might be less comparable. Hence, that may affect the reference value of these studies. Our study not only distinguished different treatment schemes of CH and Western medicine, but also limited the intervention time of included researches to 3–6 months or 12–24 weeks. In addition, we thoroughly searched eight electronic databases to ensure that we did not omit any literature that might meet the inclusion criteria. Traditional meta-analysis is a direct comparison method, and it could not compare the data of relevant clinical trials indirectly. In our study, NMA was used for the first time to compare the efficacy of CH, CH + NAC, CH + PFD, NAC, PFD and NAC + PFD directly and indirectly to search for a more reasonable treatment scheme. Therefore, our study has more advantages than previous studies to make better clinical decisions.

However, our research also has limitations. Of the 23 RCTs included, 14 ones specifically described the generation method of random sequence. All studies did not describe concealment of the distribution plan, blinding of subjects and researchers, as well as blinding of the evaluators to the outcome. Therefore, the quality of included studies is not as high as expected. Limitation of sample size in some groups may have an impact on our results and publication bias results may also be related to that. All subjects were from China and that means our conclusion might not apply to populations from other regions. Besides, the measurements of 6WMD and SGRQ are significantly related to the subjectivity of patients and testers, which may also cause bias and heterogeneity. Meanwhile, our study included only 6 treatment schemes, and thus our conclusion still has limitation. More treatment schemes need to be included in future research. Due to the short trial period we included in the study, most researches included did not evaluated indicators such as mortality, progression-free survival and acute exacerbation. In addition, the drug doses in the included RCT studies were not exactly the same, and that may also affect the accuracy of our results. Therefore, in the future, we need large sample sizes and long term RCTs which are more rational and scientific to further verify our conclusions.

## Conclusions

NMA showed that there were significant differences in terms of DLCO (%) and SGRQ between CH and NAC, while others were not. CH + NAC is the best in terms of FVC (%), SGRQ, PaO2 and PaCO2; CH is the best in terms of DLCO (%), VC (%) and 6MWD; CH + PFD is the best in terms of FVC (L). In conclusion, Chinese herbs for supplementing qi and activating blood circulation (CH) related treatments may have advantages in the treatment for IPF and Chinese herbs for supplementing qi and activating blood circulation combined with NAC (CH+NAC) may have clinical application value. However, limited by the quality and scale of researches included, more rational and scientific RCTs containing large sample sizes need to be conducted to further verify our conclusions.

## Supporting information

S1 Fig The heterogeneity of data in DLCO (%). (TIF)

S2 Fig The heterogeneity of data in VC (%). (TIF)

S3 Fig The heterogeneity of data in FVC (%). (TIF)

S4 Fig The heterogeneity of data in FVC (L). (TIF)

S5 Fig The heterogeneity of data in SGRQ. (TIF)

S6 Fig The heterogeneity of data in 6MWD. (TIF)

S7 Fig The heterogeneity of data in PaO2. (TIF)

S8 Fig The heterogeneity of data in PaCO2. (TIF)

S1 Table The rank probability of each intervention in different outcomes. (XLSX)

S2 Table PRISMA checklist. (DOC)
