# Supplementary figures and images for: Clinical efficacy of Chinese herbs for supplementing qi and activating blood circulation combined with N-acetylcysteine in the treatment of idiopathic pulmonary fibrosis: A systematic review and network meta-analysis
Source: PLoS One. 2022 Mar 4;17(3):e0265006. doi: 10.1371/journal.pone.0265006 (PMC8896725; doi:10.1371/journal.pone.0265006)

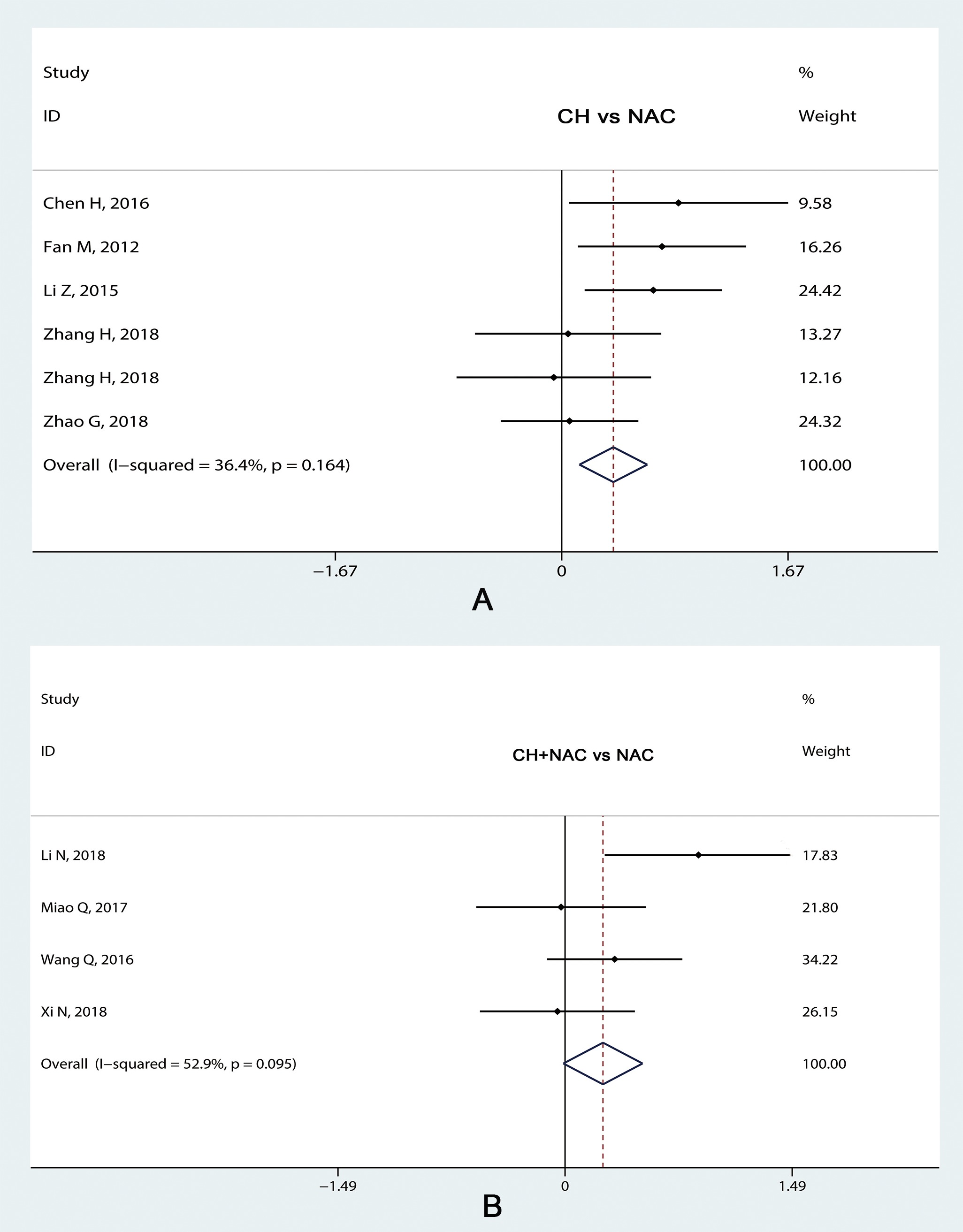

Supplement: S1 Fig — (TIF) [file pone.0265006.s001.tif]

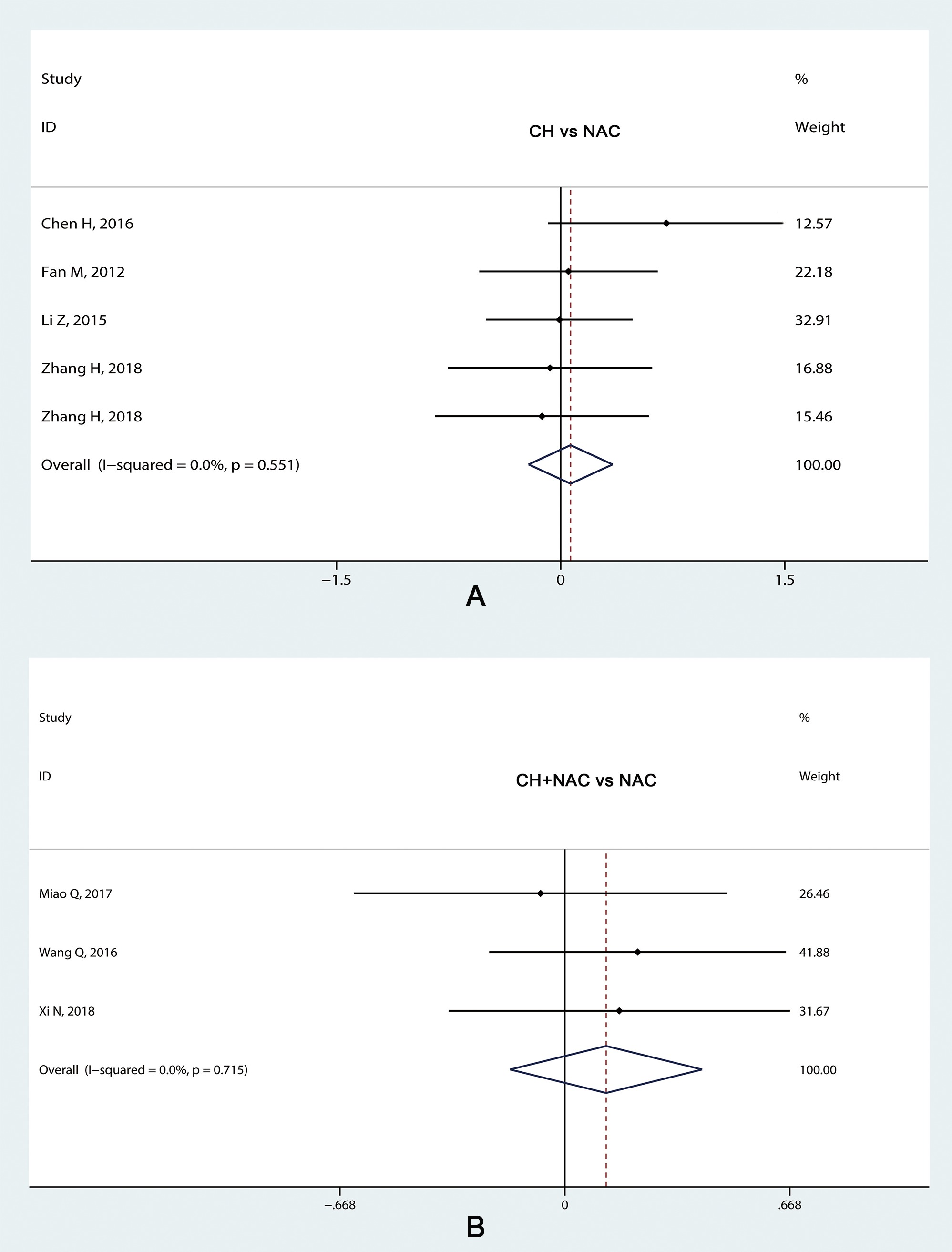

Supplement: S2 Fig — (TIF) [file pone.0265006.s002.tif]

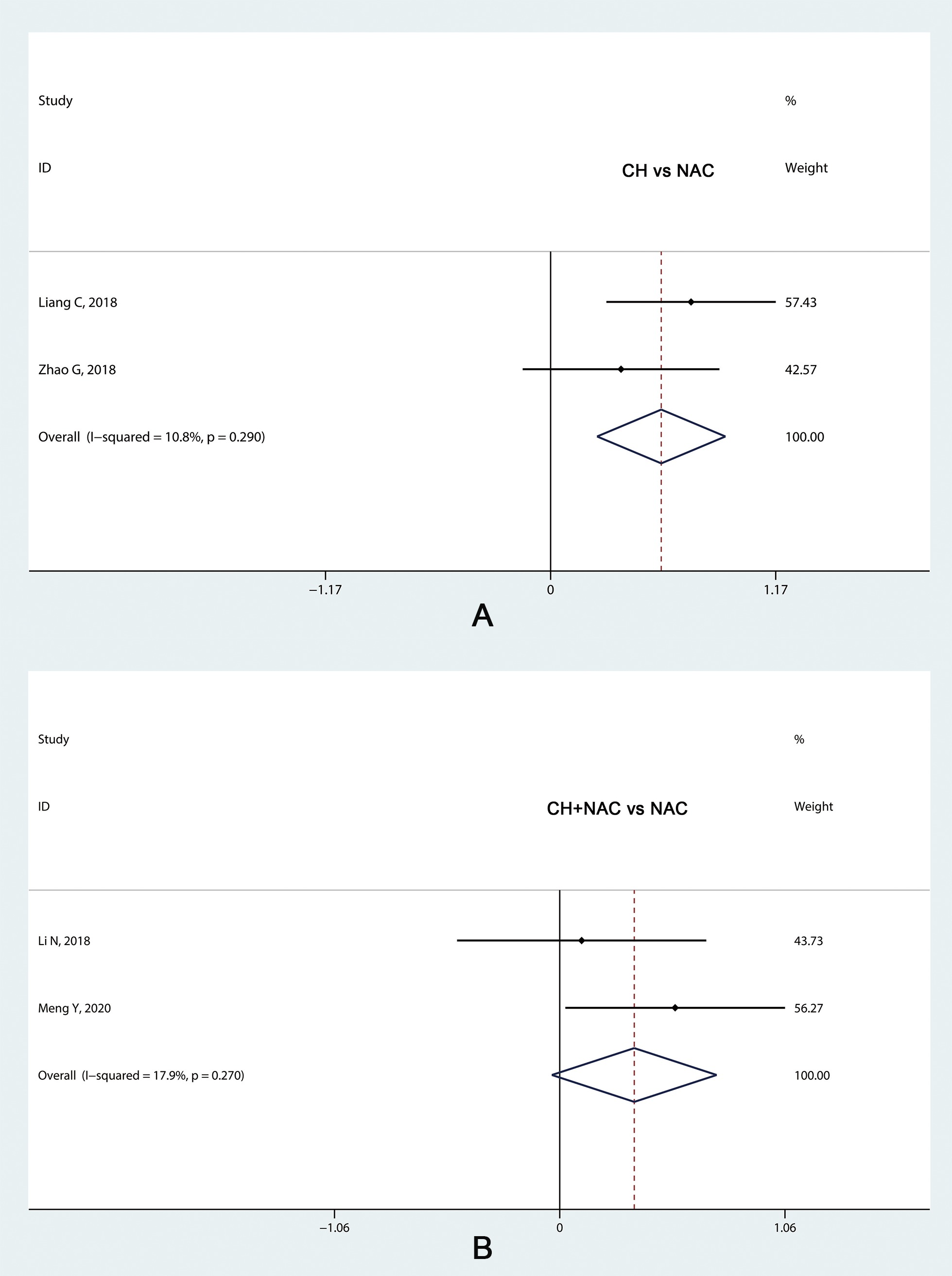

Supplement: S3 Fig — (TIF) [file pone.0265006.s003.tif]

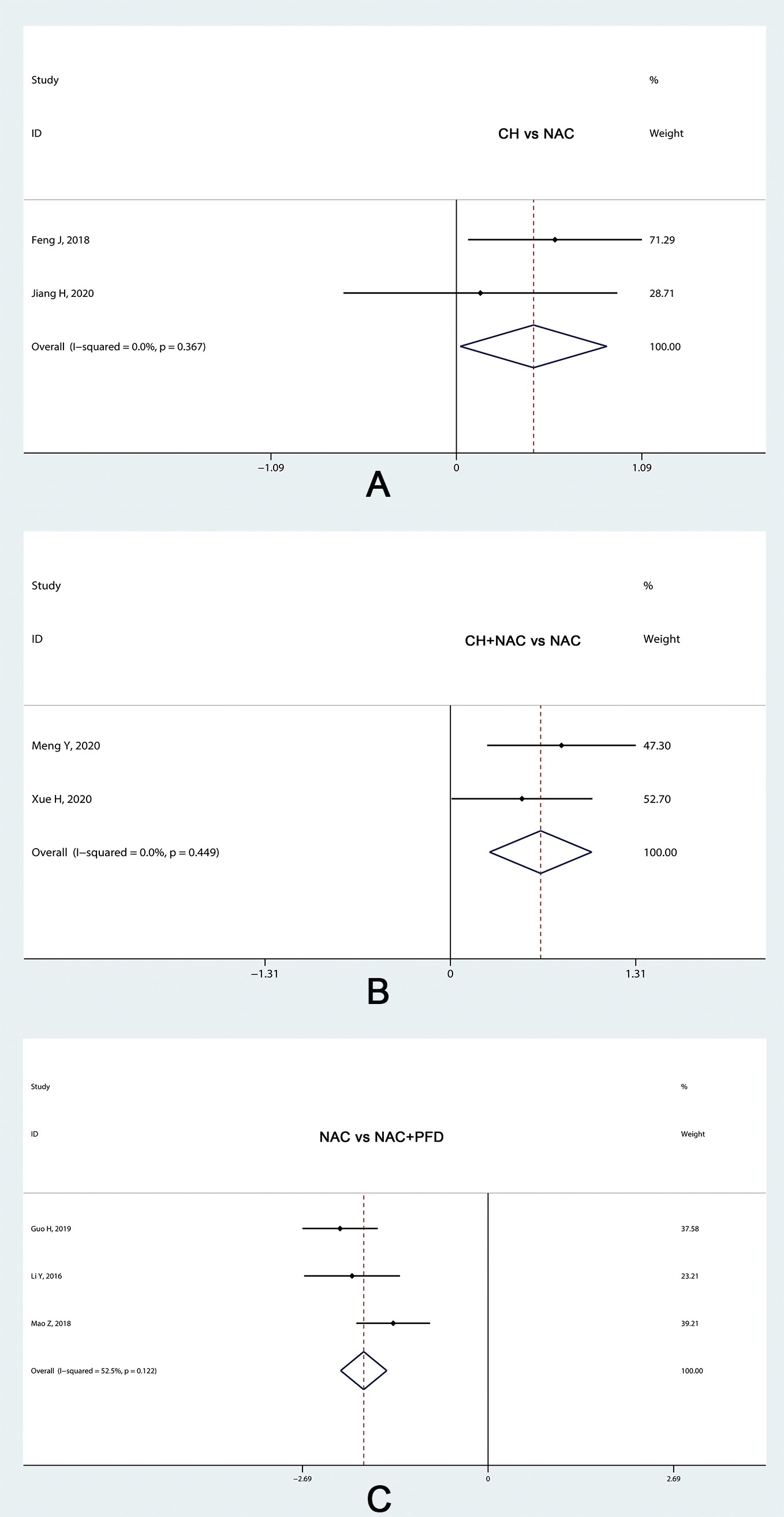

Supplement: S4 Fig — (TIF) [file pone.0265006.s004.tif]

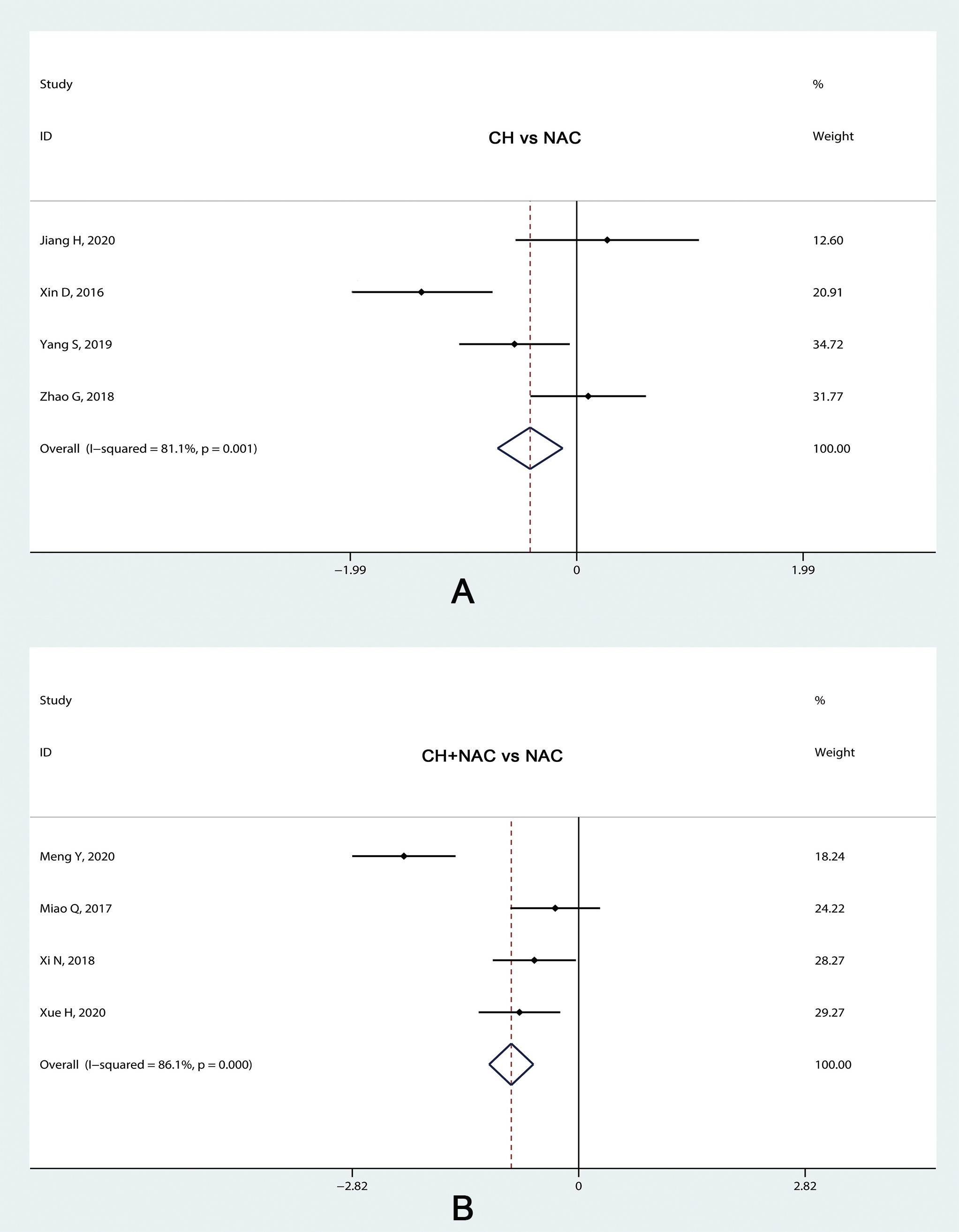

Supplement: S5 Fig — (TIF) [file pone.0265006.s005.tif]

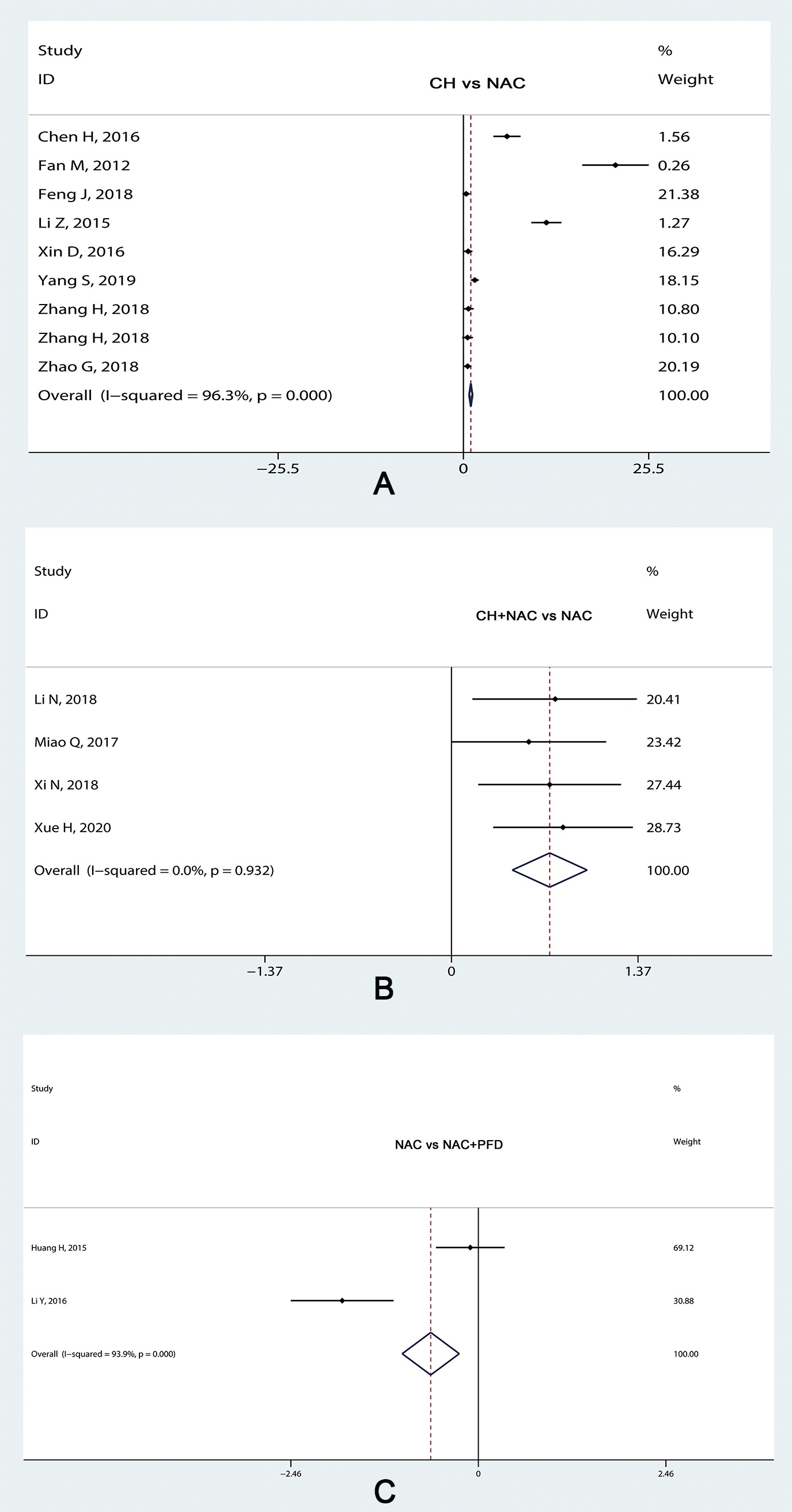

Supplement: S6 Fig — (TIF) [file pone.0265006.s006.tif]

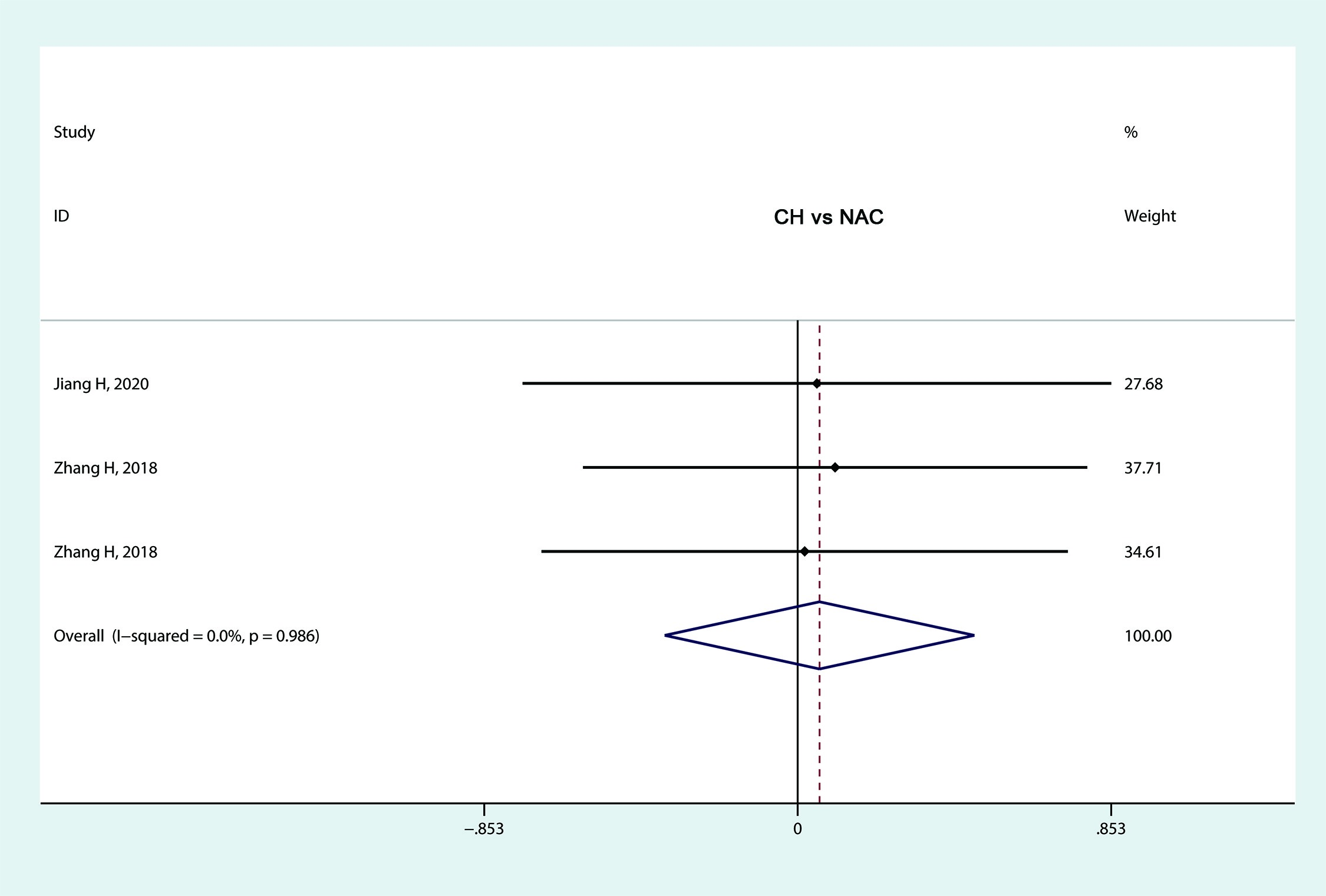

Supplement: S7 Fig — (TIF) [file pone.0265006.s007.tif]

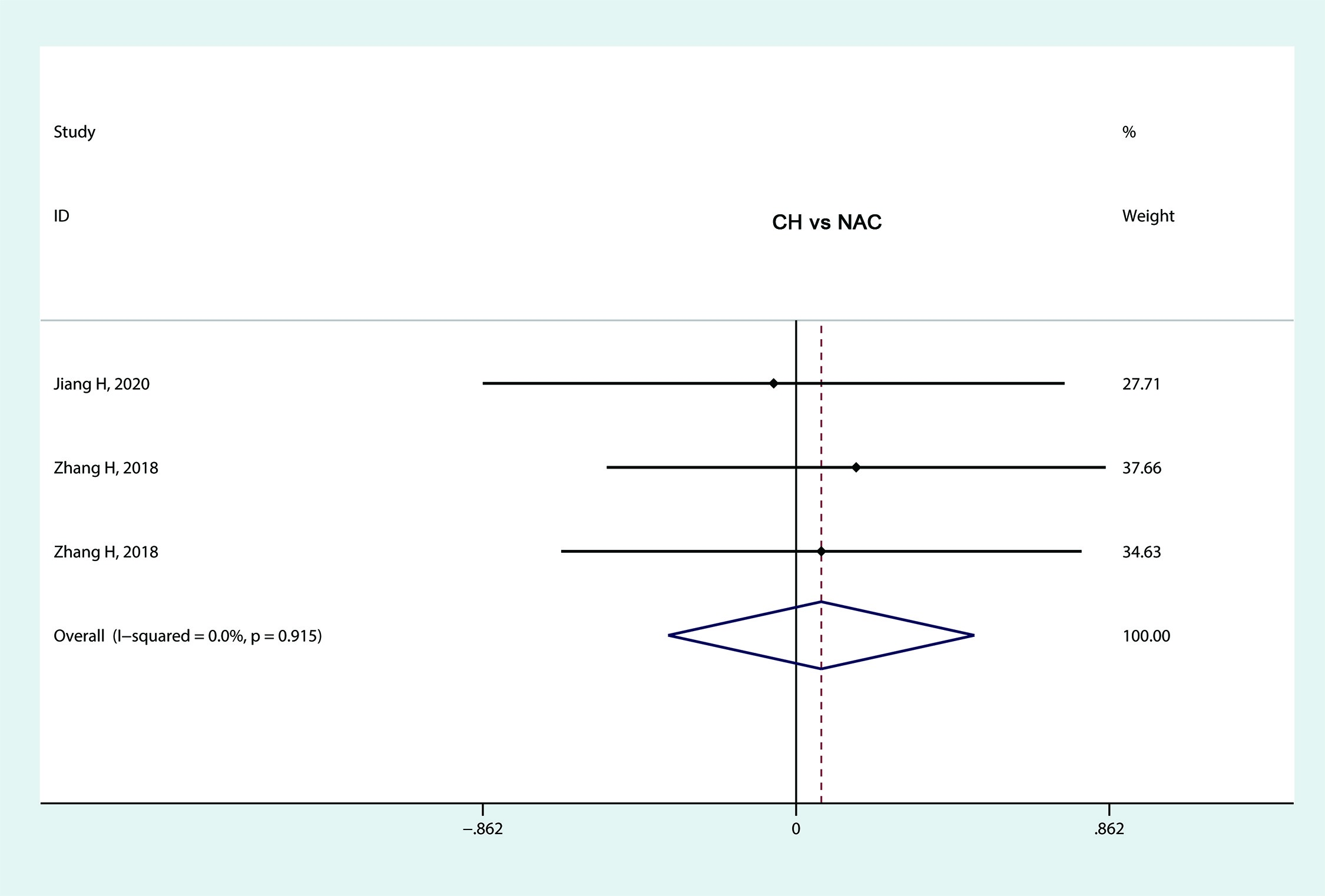

Supplement: S8 Fig — (TIF) [file pone.0265006.s008.tif]
